# Editorial for the Special Issue: “Current Technique for Antibiotic Susceptibility Test: Advantages and Limitations; Need for Next-Generation Test”

**DOI:** 10.3390/antibiotics12040750

**Published:** 2023-04-13

**Authors:** Eleonora Nicolai

**Affiliations:** Biochemistry Laboratory, Department of Experimental Medicine, University of Rome “Tor Vergata”, 00133 Rome, Italy; nicolai@med.uniroma2.it

The overuse or misuse of antibiotics, either when recommended by physicians or administered through self-medication at the time of infection, has caused drug-resistant pathogens to become a major healthcare issue, with millions of reported cases every year.

A recent report on the casualties related to antibiotic resistance released by the World Health Organization (WHO) confirmed that antibiotic resistance will soon be the most prevalent cause of death across the globe.

The rapid, sensitive detection of pathogenic bacteria could be critical for initiating timely treatment with proper antibiotics, preventing the spread of disease, and identifying infection sources in hospitals, homes, and other field settings. Accordingly, a rapid antimicrobial susceptibility test (AST) is urgently needed for the timely treatment of patients. Advanced diagnostics would allow clinicians to determine the most effective treatment more quickly, reduce the nonspecific use of broad-spectrum antimicrobials, and facilitate enrollment in new antibiotic treatments.

Although bacterial culture is the clinical gold standard, it has drawbacks, including long processing times (up to several days), high personnel costs, and the need for specialized equipment and species-specific protocols. Technical challenges, however, still remain with regard to the translation of new tests into routine clinical workflows. System operation must be simple, without requiring the assistance of trained operators, and assay costs must be lower than those for conventional screening [1,2].

A rise in antibiotic resistance is a certainty; therefore, we must develop technologies that will permit rapid AST (within few hours) and that are non-invasive (saliva- or urine-based) or minimally invasive.

Rapid diagnostics plays a pivotal role in the treatment of bacterial infections, and so does knowledge of the general biological mechanisms that regulate interactions between bacteria and cells/drugs or specific biochemical mechanisms such as those related to enzyme membrane-binding activity [3,4]. Antimicrobial resistance involves various challenges and strategies, some of which are described in the papers included in this Special Issue.

For this Special Issue, experts working on antibiotic resistance from both biological and diagnostics perspectives were invited in order to trace the prevalence of antibiotic-resistant bacteria and to propose new diagnostic solutions with which to counteract the spread of these public health burdens. 

This Special Issue includes full research articles, a review, and a prospective study. 

Among the first type of contributions, Ersoy Selvi C. and coworkers aimed to combine current AST methods with targeted genetic sequencing to identify methicillin-resistant Staphylococcus aureus that may potentially respond to standard β-lactam therapy in vivo [5]. Through a diagnostics perspective, Nicolai E. et al. presented a rapid, point-of-care, phenotypic AST device that can report the antibiotic susceptibility/resistance of a uropathogen to a panel of antibiotics in as few as 3 h via fluorescent-labelling chemistry and a highly sensitive particle-counting instrument. Unlike culture-based AST, the device presented therein can provide objective, quantitative information to the end user and is not vulnerable to inaccurate or subjective interpretations due to variables such as user-dependent differences in sample preparations, plating techniques, or zone diameter measurements [6].

Kowalski R.P presented a perspective study on eye infections [7]. Reporting on cases of eye infections, this paper suggests potential differences that must be considered when dealing with different tissues. 

Gajic I. et al. published a review on antimicrobial susceptibility testing, highlighting how the implementation of novel devices would enable the identification of optimal treatment approaches and the surveillance of antibiotic resistance in health, agriculture, and the environment, thereby facilitating monitoring programs and more effectively combatting the emergence of antimicrobial resistance [8].

Depka D. and coauthors examined the molecular basis for the carbapenem resistance mechanism and estimated the usefulness of conventional PCR and real-time PCR for the detection of oxacillinases when compared to phenotypic carbapenemase detection [9].

Wolf Lena J. focused on anaerobic bacteria, for which resistance is increasing worldwide. Their study aimed to provide susceptibility data for rare anaerobes and compare the gold-standard agar dilution method to the time-saving E-test methodology. Consequently, even though the species that were tested in this study are rare, it was unclear whether E-tests provided reliable results [10].

Sparbrod M. and co-workers focused on the analysis of subgingival biofilm samples from periodontitis patients, investigating the relationship between phenotypic and genotypic resistance. The authors concluded that antibiotic resistance may be polygenetic, and genes may be silent [11]. In relation to the need for and lack of rapid bacterial diagnostics, the work of Malita M.A. demonstrated how an annual report that monitors antimicrobial resistance trends in health care facilities may provide a profile of empirical therapies useful in diverse emergency situations, such as the transmission of resistant bacteria to the oral cavity of newborn babies [12]. The final study included in this collection is a paper by Cerini P. et al. that compared the trends in antimicrobial resistance in clinical isolates, divided by year and by bacteria spp., of samples obtained from nosocomial and community patients. Italian patient records were collected from 2019 to 2022. An increase in the prevalence of several antibiotic-resistant bacteria was observed for both community and nosocomial patients. This work sounds an alarm bell highlighting the necessity of adopting preventive and control measures to reduce the spread of multidrug-resistant pathogens [13].
